# Early Life Stress, Nicotinic Acetylcholine Receptors and Alcohol Use Disorders

**DOI:** 10.3390/brainsci5030258

**Published:** 2015-06-30

**Authors:** Joan Y. Holgate, Selena E. Bartlett

**Affiliations:** Institute of Health and Biomedical Innovation, Translational Research Institute, Queensland University of Technology, 37 Kent St, Woolloongabba, Queensland 4102, Australia; E-Mail: Selena.bartlett@qut.edu.au

**Keywords:** Early life stress, alcohol, nicotinic acetylcholine receptors, stress resilience, nucleus accumbens, cholinergic, mesolimbic, dopamine, GABA

## Abstract

Stress is a major driving force in alcohol use disorders (AUDs). It influences how much one consumes, craving intensity and whether an abstinent individual will return to harmful alcohol consumption. We are most vulnerable to the effects of stress during early development, and exposure to multiple traumatic early life events dramatically increases the risk for AUDs. However, not everyone exposed to early life stress will develop an AUD. The mechanisms determining whether an individual’s brain adapts and becomes resilient to the effects of stress or succumbs and is unable to cope with stress remain elusive. Emerging evidence suggests that neuroplastic changes in the nucleus accumbens (NAc) following early life stress underlie the development of AUDs. This review discusses the impact of early life stress on NAc structure and function, how these changes affect cholinergic signaling within the mesolimbic reward pathway and the role nicotinic acetylcholine receptors (nAChRs) play in this process. Understanding the neural pathways and mechanism determining stress resilience or susceptibility will improve our ability to identify individuals susceptible to developing AUDs, formulate cognitive interventions to prevent AUDs in susceptible individuals and to elucidate and enhance potential therapeutic targets, such as the nAChRs, for those struggling to overcome an AUD.

## 1. Alcohol Use Disorders: What’s All the Stress About?

Alcohol use disorders (AUDs) constitute a major global health issue and there remains a critical need for the development of medications for the treatment of AUDs. Stress is a significant contributing factor in AUDs [1,2,3] and the ability to cope with stress (known as resilience) inversely predicts the development of a stress-related neuropsychiatric disease, including AUDs [4]. Susceptibility to AUDs is determined by both genetic and environmental factors [1,5,6]. However, chronic exposure to an adverse environment dramatically increases the risk toward developing AUDs [6,7,8]. Research indicates that this is not a passive process; that individuals are able to learn to be resilient by developing protective mechanisms that shield them from the maladaptive effects of stress [4]. Early life stress (ELS) has been identified as a significant factor contributing to the development of numerous stress-related psychiatric disorders [1,4,5,6,9,10]. Children with a family history of alcoholism are particularly vulnerable to developing psychiatric disorders later in life. Their family history of alcoholism not only increases their risk of developing AUDs: it places them at an increased risk for exposure to an aversive environment in early life [7,11]. For these children it is a vicious cycle as exposure to multiple traumatic early life events increases the risk of developing AUDs approximately seven-fold [7]. Understanding the neural pathways involved and the mechanism that determine resilience or susceptibility to the effects of stress will improve our ability to identify individuals susceptible to developing AUDs, formulate cognitive interventions to prevent the development of AUDs in susceptible individuals and to elucidate and enhance potential therapeutic targets such as the nicotinic acetylcholine receptors (nAChRs) for those already struggling to overcome an AUD.

## 2. The Two-Way Interplay Between Stress and Alcohol Controls Alcohol Consumption

Stress is a major driving force in AUDs [1,2,3,5,12,13,14,15]. It influences how much alcohol an individual consumes (for review see [16]), how intensely one craves alcohol (for reviews see [17,18]) and ultimately whether an abstinent individual will return to harmful alcohol consumption [2,16,17,18,19,20]. Additionally, the chronic consumption of alcohol alters the normal function of the stress system causing an increased susceptibility to stress [19]. This has devastating consequences for the progression of AUDs as it produces a cycle of degeneration where exposure to stress leads to escalations in alcohol consumption, further reducing the ability to cope with stress and shortening the length of intervals between periods of abstinence.

## 3. Stress Changes How the Brain Functions

**Figure 1:**
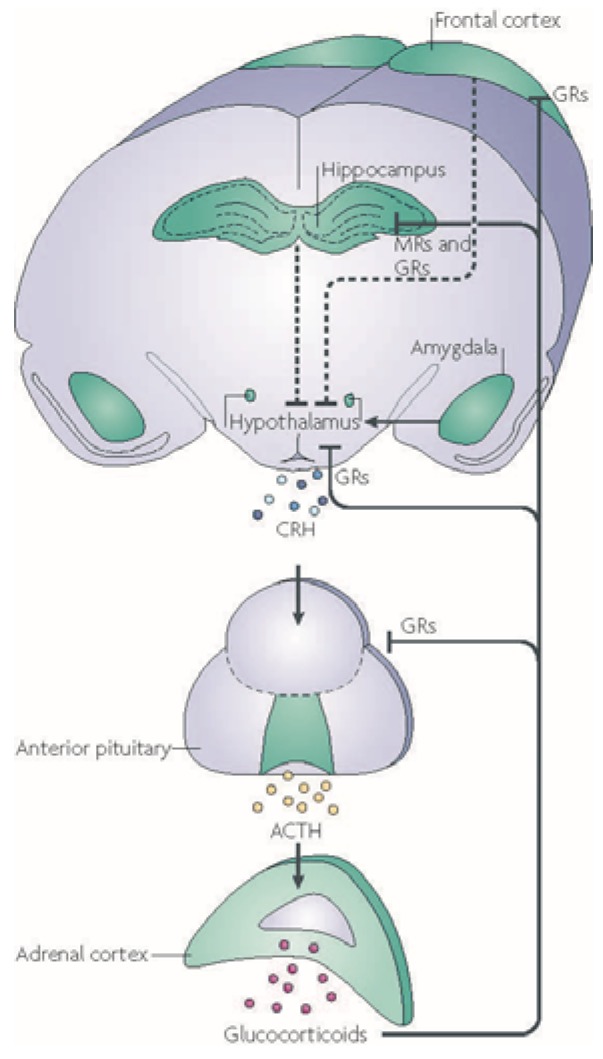
The stress response. Stressful events trigger the release of corticotrophin releasing hormone (CRH) from the hypothalamus which results in the release of adrenocorticotropic hormone (ACTH) from the pituitary into the blood. This causes glucocorticoids to be released from the adrenals which bind to glucocorticoid (GRs) and mineralocorticoid (MRs) receptors creating a negative feedback circuit, ending the stress response and restoring allostasis. Adapted from [22].

The mechanisms underlying the brain’s response to stress are well understood. In the brain stressful events trigger the release of corticotrophin releasing hormone (CRH) and vasopressin from the paraventricular nucleus of the hypothalamus. This causes the pituitary to secrete adrenocorticotropic hormone (ACTH), resulting in the release of glucocorticoids (primarily cortisol in humans, corticosterone in rodents) from the adrenals (Figure 1) [16,21].

Although there are many different types of receptors in the brain that mediate alcohol consumption and seeking behaviours (for reviews see [23,24,25]), it is currently presumed that glucocorticoids do not act directly at these receptors. Rather, it is proposed that glucocorticoids, via mineralocorticoid (MR) and glucocorticoid (GR) receptors, alter the activity and excitability of neurons by facilitating or inhibiting the signaling of ion channels, receptors and neurotransmitters and the consumption of alcohol relieves the effects of the alterations caused by stress (for review see [21]). Research indicates that MRs play a prominent role in acute stress responses as they act to maintain allostasis (allostasis is the process of maintaining stability, or homeostasis, during change (see [26,27])). GRs, on the other hand act during chronic stress, where allostasis cannot be restored and instead the system must adapt to the new environment by using inefficient stress response processes (known as allostatic load) (for reviews see [21,27,28]). Despite the cost of maintaining allostatic load, the actions of glucocorticoids and GR can remain protective, promoting neuroplastic changes with positive effects. However, when the system becomes overrun (allostatic overload) the actions of the glucocorticoids become damaging and the GR-mediated changes in gene transcription, chemical signaling and brain morphology lead to disease such as depression and AUDs [28].

It is clear from human studies that not everyone who experiences stress will become an alcoholic; stress-induced increases in alcohol intake are limited to alcohol-dependent individuals and individuals demonstrating traits associated with elevated stress (anxiety and depression) [29,30]. We know these differences in susceptibility to stress-related disorders result from a complex interaction of the individuals’ genetics and life experiences (for reviews see [6,31,32]). Additionally we know that stressful experience(s) in early life plays an important role in this interaction [5,6,33]; but the exact mechanisms determining stress resilience or susceptibility have remained elusive. In recent years there is an increasing number of studies that suggest neuroplastic changes within the nucleus accumbens (NAc) following exposure to ELS may underlie the development of numerous neuropsychiatric disorders including AUDs [34,35,36,37,38,39,40,41,42,43,44,45,46].

## 4. AUDs and the Nucleus Accumbens

Alcohol changes the function of the NAc. Rodent studies have shown that exposure to alcohol enhances activation of the NAc [47,48], alters NAc dopamine [49] and glutamate [50,51,52] transmission and modifies dendritic structure [53]. These studies also show that ethanol has differential effects on the NAc core and the shell. In the NAc shell ethanol alters dendrite morphology [54], cFos expression [55] and gamma amino butyric acid (GABA) [56] and dopamine [57] signaling. Whereas, in the NAc core ethanol exposure alters dendrite morphology [53], glutamate signaling [51,52,53] and mitogen-activated protein kinase (MAPK) expression [47]. In humans, a family history of alcoholism is associated with altered NAc volume and NAc functional connectivity. Consistent with studies showing that females are more vulnerable to the effects of ELS [40,58,59,60] and two times more likely to develop AUDs following ELS [7], a link between altered left NAc volume and a family history of alcoholism has been reported for adolescent females but not males [61]. Human studies into schizophrenia show similar disruptions in NAc-prefrontal cortex (PFC) connectivity and suggest that changes in the NAc shell may mediate the positive symptoms associated with schizophrenia (For reviews see [46,62,63,64]).

## 5. Early Life Stress Causes Neuroplastic Changes in the Nucleus Accumbens

Exposure to ELS also impacts the function of the NAc. In rodents, exposure to ELS alters dopamine [34,65,66] and serotonin signaling in the NAc [67,68]. Both neurotransmitters modulate relapse to alcohol seeking [25,67,69]. Changes in expression of genes and proteins involved in the stress response, like GRs and corticotrophin releasing hormone (CRH) receptors have also been found following exposure to ELS [70,71]. In humans, exposure to ELS has been linked to reduced NAc reactivity [36]. This contradicts the popular hypothesis that the positive symptoms of schizophrenia are due to reduced GABA-mediated inhibition of the NAc [46]. Differences in sex and the type and number of exposures to ELS may account for the discrepancies in these findings.

Research into the effects of ELS on the NAc shell and core is still in its early stages. Enhancement of estrogen, oxytocin and serotonin-1A receptor expression have been found in the NAc shell following exposure to short periods of maternal separation in female rodents [67]. This type of ELS is thought to model stress resilience [10,72,73]. In females, the interaction of these three receptors is proposed to be critical in the development of anxiety and depression disorders [68,74,75]. In the NAc core reductions of methyl CpG binding protein 2 (MeCP2) were found following ELS in rodents [39]. MeCp2 is commonly used as an epigenetic marker. A growing number of studies have provided evidence suggesting that ELS causes epigenetic changes in gene transcription of the GRs contributing to disruptions in the mesolimbic pathway [76,77,78,79,80,81,82,83,84,85,86,87,88]. Much more research is required to further elucidate the roles of the NAc core and shell in the development of stress resilience and susceptibility and how this contributes to the development of AUDs.

## 6. Nucleus Accumbens Regulates Cholinergic Output to PFC

The NAc modulates the activity of the basal forebrain which is the major projection site for cholinergic neurons within the brain [46,62,89,90,91,92]. The basal forebrain cholinergic neurons project to most of the cortex, including the PFC. Changes in cholinergic output to the PFC (which is responsible for differentiating between conflicting thoughts, like good vs bad, prediction of future consequences and urge suppression) have been proposed to underlie the symptoms of numerous psychiatric disorders including PTSD, schizophrenia and major depression (for reviews see [41,46,62,63,64,93,94,95]). It is has been proposed that there is a reduction in GABAergic inhibition of NAc activity from the amygdala and VTA which leads to enhanced activation of the PFC (for reviews see [46,96]). The PFC in turn regulates activity of VTA and amygdala producing a generalized malfunction of mesolimbic pathway [46,91,97,98,99]. Cholinergic neurons regulate most of the mesolimbic pathway via the release of acetylcholine (ACh); including the amygdala, NAc, VTA and PFC [91,97]. ACh binds to nAChRs which are capable of modulating the release of dopamine and hence the rewarding and reinforcing properties of numerous drugs, including alcohol [57,100,101,102,103,104].

## 7. What are nAChRs and How are They Involved in AUDs?

**Figure 2:**
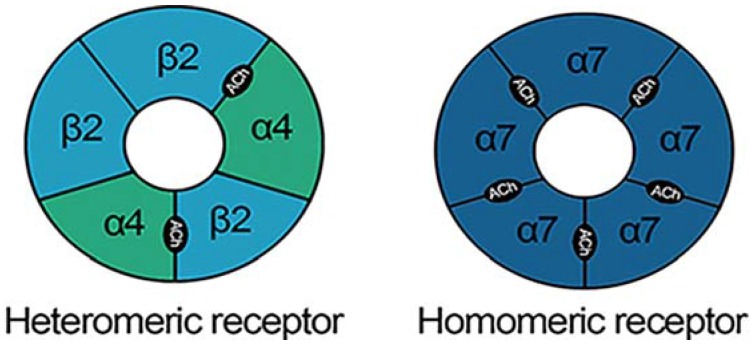
Nicotinic acetylcholine receptors (nAChRs) consist of different combinations of alpha (α) and beta (β) subunits. Variations in the subunit composition not only determine the number of binding sites for their endogenous ligand, acetylcholine (ACh) but also the functional properties of the receptor. Taken from [109].

**Figure 3:**
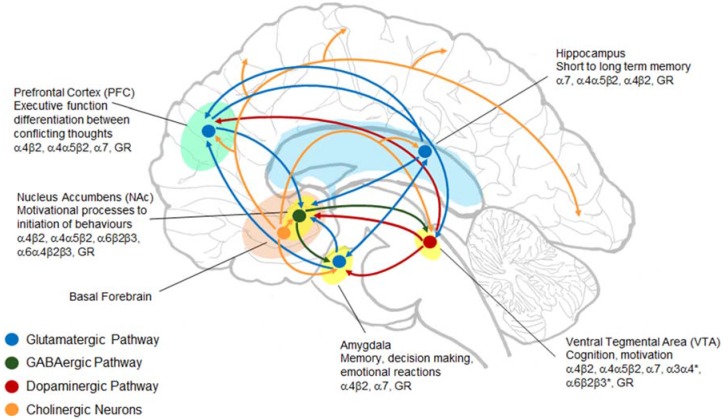
Nicotinic acetylcholine receptors (nAChRs) are located within brain structures involved in modulating alcohol addiction and stress. nAChRs are found within the mesolimbic pathway (hippocampus, prefrontal cortex (PFC), nucleus accumbens (NAc), amygdala and ventral tegmental area (VTA)). These regions also express glucocorticoid receptors (GR) and participate in glutamatergic (blue), GABAergic (green), dopaminergic (red) and cholinergic (orange) neurotransmission. Adapted from [110].

nAChRs are pentameric ligand-gated ion channels consisting of different combinations of α2–α10 and β2–β4 subunits [105] (Figure 2). Their endogenous ligand is ACh but they also bind nicotine. In a similar manner to glucocorticoids, ethanol does not modulate nAChRs directly: it instead increases the release of ACh. The type of subunits that make up the nAChR and their location in the brain influences the functional properties of the receptor. For example, in the ventral tegmental area (VTA) activation of α4/α6β4 containing (*) nAChRs modulates dopaminergic transmission whereas, α7 nAChRs modulate glutamate release and α4β2*nAChRs the release of GABA [106]. nAChRs are expressed on neurons within the mesolimbic dopaminergic pathway (Figure 3) which mediates the rewarding and reinforcing properties of ethanol [101,107,108].

Ethanol triggers the release of ACh in the VTA which causes dopamine to be released in the NAc [57,100,103,104]. The release of dopamine in this area is responsible for the mood-altering properties of ethanol: that is consumption of alcohol causes increases in dopamine making you “feel good” and decreases, like that which occurs during withdrawal, make you “feel bad” [101]. This effect is mediated by nAChRs as it can be blocked by the intra-VTA administration of mecamylamine, a non-selective nAChR antagonist [57,100,103]. The NAc responds to changes in dopamine levels by altering the activity of the cholinergic neurons of the basal forebrain, which project throughout most of the brain and heavily innervate the cortex [46,98,99,111,112]. Extra-hypothalamic structures involved in the mesolimbic dopaminergic pathway, such as the PFC, hippocampus, amygdala, NAc and VTA, also modulate the stress-HPA axis and are innervated by basal forebrain cholinergic neurons (Figure 3).

## 8. Nicotinic Acetylcholine Receptors are Modulated by Alcohol and Stress

The cholinergic system plays an important role in mediating AUDs (for reviews see [113,114]). Alcohol consumption and withdrawal affect ACh release in the brain [115,116] and compounds which alter the function of nicotinic acetylcholine receptors (nAChRs) reduce alcohol consumption and reinstatement of ethanol seeking [117,118,119,120]. It is also well known that stress alters the function of the cholinergic system [1,121,122]. This is not surprising given that ACh primarily acts as a neuromodulator, altering the state of neurons in response to changing environmental stimuli (for review see [97]) similar to glucocorticoids in response to stressful events. We also know that nAChRs are involved in the stress response. Mecamylamine, a non-selective nAChR antagonist, prevents CRH-induced increases in plasma corticosterone [123]. It also prevents nicotine-induced increases in urinary corticosterone [124] and stress-induced reinstatement of conditioned place preference to ethanol [20]. However, it is unknown which types of nAChRs and how their location within the brain modulates this process.

## 9. nAChR Subtypes and Their Role in Stress and AUDs

If you consider that ACh is a neuromodulator and nAChRs are located in the mesolimbic and stress-HPA axis systems, the cholinergic system is ideally situated for modulating alcohol consumption and relapse in response to stress. However; the precise role of the individual subunits comprising nAChRs involved in this process and the importance of their location remained relatively unexplored until recently. There is growing evidence suggesting the α4 subunit plays a prominent role in alcohol consumption driven by stress. Human genetic studies have indicated that mutations in the CHRNA4 gene, encoding the α4 subunit, is linked to a vulnerability to both alcoholism [125] and depression [121]. It has also been shown that TC-2559, an α4β2*nAChR selective agonist, increases urinary corticosterone [124]; varenicline, a partial agonist at α4β2*nAChRs, reduces ethanol consumption [119]; and prenatal stress alters α4β2*nAChR expression in the hippocampus [126]. Additionally our laboratory has shown that exposure to ethanol alters α4*nAChR expression in the NAc, amygdala and VTA of mice (unpublished data).

While circumstantial, this evidence suggests that α4*nAChRs could be an important link between AUDs and stress. Interestingly, human studies have found an association between a family history of alcoholism and: left NAc volume in adolescent females [61]; resting state connectivity of the NAc [127]; and NAc connectivity during reward [128,129] suggesting these individuals have less segregation between the NAc and executive functioning brain regions (like the PFC), and less integration with reward-related brain areas (like the amygdala and VTA). As previously discussed all these brain regions are innervated by cholinergic neurons and contain nAChRs with α4 subunits. Changes in NAc activity were also found following recent negative life stress in individuals with major depressive disorder [37]. Furthermore, polymorphisms in the CHRNA4 gene have been linked to major depression [121] and negative emotionality [130]. While it seems highly likely that the NAc modulates stress-driven alcohol consumption and relapse via α4*nAChRs, it is however difficult to determine whether the α4 subunit is acting alone or in combination with the β4 subunit as the studies discussed above do not explore this possibility. This may be due to the technical difficulties involved separating the functional properties of the individual subunits and the fact that both subunits tend to be expressed together within the brain. Recent advances in transgenic technology utilizing fluorescent tags attached to the various nAChR subunits have the potential for isolating the roles of the individual subunits in this process.

While it has been established that α4*nAChRs are important in AUDs [117,119,125], recent research indicates that there are other subtypes involved. A study by Chatterjee et al (2011) [118] shows that pharmacological modulation of α3β4*nAChRs reduces ethanol consumption in rats. More recently Cippitelli et al (2015) [131] confirmed this finding by demonstrating that pharmacological modulation of α3β4*nAChRs reduces ethanol consumption and blocks stress-induced but not cue-induced reinstatement to ethanol seeking, suggesting that the partial α3β4*nAChR agonist (AT-1001) is alleviating the effects of stress rather than the effects of ethanol. Homomeric α7 nAChRs may also be important modulators of stress and alcohol consumption. Expression of α7 nAChRs are altered in the frontal cortex and hippocampus following exposure to prenatal stress [132,133]. Additionally, a selective α7 nAChR partial agonist SSR180711, administered *ex vivo* caused an increase in dopamine in the PFC [134]. The effect of SSR180711 was blocked when the selective α7 nAChR antagonist, methyllycaconitine was employed. Changes in nAChR-mediated dopamine signaling in PFC has the potential to alter the activity of the NAc, amygdala and basal forebrain leading to changes in alcohol craving in response to stress. Interestingly, α7 nAChRs have been implicated in alcohol consumption, ELS and schizophrenia [126,133,134,135]. However, the role of these and other nAChR subtypes in stress resilience and susceptibility following ELS remains relatively unexplored.

## 10. Conclusions

While the role of nAChRs is well established in ethanol consumption and relapse, much more research is required to elucidate the contribution the various nAChR subtypes make in the development of AUDs. Even less is known about their role in stress and stress resilience and how this impacts both the development and progression of AUDs. Emerging evidence indicates that NAc-mediated changes in the cholinergic output from the basal forebrain following exposure to ELS play a critical role in the development of AUDs (and other disorders) later in life. However, it remains to be determined what role the nAChRs play in this process. Gaining greater insight into the role of nAChRs in stress resilience will further our ability to identify individuals at risk of developing AUDs, prevent the development of AUDs in those at risk and develop better pharmacotherapeutics to treat those struggling with an AUD.

## References

[1] Srinivasan S., Shariff M., Bartlett S.E. (2013). The role of the glucocorticoids in developing resilience to stress and addiction. Front. Psychiatry.

[2] Sinha R., Jastreboff A.M. (2013). Stress as a common risk factor for obesity and addiction. Biol. Psychiatry.

[3] Haass-Koffler C.L., Bartlett S.E. (2012). Stress and addiction: Contribution of the corticotropin releasing factor (crf) system in neuroplasticity. Front. Mol. Neurosci..

[4] Russo S.J., Murrough J.W., Han M.H., Charney D.S., Nestler E.J. (2012). Neurobiology of resilience. Nat. Neurosci..

[5] Enoch M.A. (2011). The role of early life stress as a predictor for alcohol and drug dependence. Psychopharmacology.

[6] Enoch M.A. (2012). The influence of gene-environment interactions on the development of alcoholism and drug dependence. Curr. Psychiatry Rep..

[7] Brady K.T., Back S.E. (2012). Childhood trauma, posttraumatic stress disorder, and alcohol dependence. Alcohol Res. Curr. Rev..

[8] Daoura L., Haaker J., Nylander I. (2011). Early environmental factors differentially affect voluntary ethanol consumption in adolescent and adult male rats. Alcohol. Clin. Exp. Res..

[9] Anisman H., Zaharia M.D., Meaney M.J., Merali Z. (1998). Do early-life events permanently alter behavioral and hormonal responses to stressors?. Int. J. Dev. Neurosci..

[10] Levine S. (2005). Developmental determinants of sensitivity and resistance to stress. Psychoneuroendocrinology.

[11] Chassin L., Pillow D.R., Curran P.J., Molina B.S., Barrera M. (1993). Relation of parental alcoholism to early adolescent substance use: A test of three mediating mechanisms. J. Abnorm. Psychol..

[12] Contoreggi C., Lee M.R., Chrousos G. (2013). Addiction and corticotropin-releasing hormone type 1 receptor antagonist medications. Ann. NY Acad. Sci..

[13] See R.E., Waters R.P. (2010). Pharmacologically-induced stress: A cross-species probe for translational research in drug addiction and relapse. Am. J. Transl. Res..

[14] Shalev U., Erb S., Shaham Y. (2010). Role of crf and other neuropeptides in stress-induced reinstatement of drug seeking. Brain Res..

[15] Simms J.A., Nielsen C.K., Li R., Bartlett S.E. (2014). Intermittent access ethanol consumption dysregulates crf function in the hypothalamus and is attenuated by the crf-r1 antagonist, cp-376395. Addict. Biol..

[16] Spanagel R., Noori H.R., Heilig M. (2014). Stress and alcohol interactions: Animal studies and clinical significance. Trends Neurosci..

[17] Breese G.R., Sinha R., Heilig M. (2011). Chronic alcohol neuroadaptation and stress contribute to susceptibility for alcohol craving and relapse. Pharmacol. Ther..

[18] Haass-Koffler C.L., Leggio L., Kenna G.A. (2014). Pharmacological approaches to reducing craving in patients with alcohol use disorders. CNS Drugs.

[19] Adinoff B., Iranmanesh A., Veldhuis J., Fisher L. (1998). Disturbances of the stress response: The role of the hpa axis during alcohol withdrawal and abstinence. Alcohol Health Res. World.

[20] Bhutada P., Mundhada Y., Ghodki Y., Dixit P., Umathe S., Jain K. (2012). Acquisition, expression, and reinstatement of ethanol-induced conditioned place preference in mice: Effects of exposure to stress and modulation by mecamylamine. J. Psychopharmacol. (Oxf., Engl.).

[21] Groeneweg F.L., Karst H., de Kloet E.R., Joels M. (2011). Rapid non-genomic effects of corticosteroids and their role in the central stress response. J. Endocrinol..

[22] Lupien S.J., McEwen B.S., Gunnar M.R., Heim C. (2009). Effects of stress throughout the lifespan on the brain, behaviour and cognition. Nat. Rev. Neurosci..

[23] Arias A.J., Sewell R.A. (2012). Pharmacogenetically driven treatments for alcoholism. CNS Drugs.

[24] Heilig M., Goldman D., Berrettini W., O’Brien C.P. (2011). Pharmacogenetic approaches to the treatment of alcohol addiction. Nat. Rev. Neurosci..

[25] Vengeliene V., Bilbao A., Molander A., Spanagel R. (2008). Neuropharmacology of alcohol addiction. Br. J. Pharmacol..

[26] McEwen B.S. (2000). Allostasis and allostatic load: Implications for neuropsychopharmacology. Neuropsychopharmacology.

[27] McEwen B.S. (2008). Central effects of stress hormones in health and disease: Understanding the protective and damaging effects of stress and stress mediators. Eur. J. Pharmacol..

[28] De Kloet E.R. (2004). Hormones and the stressed brain. Ann. NY Acad. Sci..

[29] Cooper M.L., Russell M., Skinner J.B., Frone M.R., Mudar P. (1992). Stress and alcohol use: Moderating effects of gender, coping, and alcohol expectancies. J. Abnorm. Psychol..

[30] Miller P.M., Hersen M., Eisler R.M., Hilsman G. (1974). Effects of social stress on operant drinking of alcoholics and social drinkers. Behav. Res. Ther..

[31] Adriana D.-A., Alejandro D.-M., Leonila Rosa D.-M. (2011). The complex interplay of genetics, epigenetics, and environment in the predisposition to alcohol dependence. Salud Ment..

[32] Zannas A.S., West A.E. (2014). Epigenetics and the regulation of stress vulnerability and resilience. Neuroscience.

[33] Roman E., Nylander I. (2005). The impact of emotional stress early in life on adult voluntary ethanol intake-results of maternal separation in rats. Stress (Amst., Neth.).

[34] Brenhouse H.C., Lukkes J.L., Andersen S.L. (2013). Early life adversity alters the developmental profiles of addiction-related prefrontal cortex circuitry. Brain Sci..

[35] Choy K.H., van den Buuse M. (2008). Attenuated disruption of prepulse inhibition by dopaminergic stimulation after maternal deprivation and adolescent corticosterone treatment in rats. Eur. Neuropsychopharmacol. J. Eur. Coll. Neuropsychopharmacol..

[36] Goff B., Gee D.G., Telzer E.H., Humphreys K.L., Gabard-Durnam L., Flannery J., Tottenham N. (2013). Reduced nucleus accumbens reactivity and adolescent depression following early-life stress. Neuroscience.

[37] Hsu D.T., Langenecker S.A., Kennedy S.E., Zubieta J.K., Heitzeg M.M. (2010). Fmri bold responses to negative stimuli in the prefrontal cortex are dependent on levels of recent negative life stress in major depressive disorder. Psychiatry Res..

[38] Karkhanis A.N., Locke J.L., McCool B.A., Weiner J.L., Jones S.R. (2014). Social isolation rearing increases nucleus accumbens dopamine and norepinephrine responses to acute ethanol in adulthood. Alcohol. Clin. Exp. Res..

[39] Lewis C.R., Staudinger K., Scheck L., Olive M.F. (2013). The effects of maternal separation on adult methamphetamine self-administration, extinction, reinstatement, and mecp2 immunoreactivity in the nucleus accumbens. Front. Psychiatry.

[40] Pena C.J., Neugut Y.D., Calarco C.A., Champagne F.A. (2014). Effects of maternal care on the development of midbrain dopamine pathways and reward-directed behavior in female offspring. Eur. J. Neurosci..

[41] Post R.M. (2010). Mechanisms of illness progression in the recurrent affective disorders. Neurotox. Res..

[42] Silveira P.P., Portella A.K., Assis S.A., Nieto F.B., Diehl L.A., Crema L.M., Peres W., Costa G., Scorza C., Quillfeldt J.A. (2010). Early life experience alters behavioral responses to sweet food and accumbal dopamine metabolism. Int. J. Dev. Neurosci..

[43] Wei Q., Fentress H.M., Hoversten M.T., Zhang L., Hebda-Bauer E.K., Watson S.J., Seasholtz A.F., Akil H. (2012). Early-life forebrain glucocorticoid receptor overexpression increases anxiety behavior and cocaine sensitization. Biol. Psychiatry.

[44] Yorgason J.T., Espana R.A., Konstantopoulos J.K., Weiner J.L., Jones S.R. (2013). Enduring increases in anxiety-like behavior and rapid nucleus accumbens dopamine signaling in socially isolated rats. Eur. J. Neurosci..

[45] Yu P., An S., Tai F., Wang J., Wu R., Wang B. (2013). Early social deprivation impairs pair bonding and alters serum corticosterone and the nacc dopamine system in mandarin voles. Psychoneuroendocrinology.

[46] Sarter M., Nelson C.L., Bruno J.P. (2005). Cortical cholinergic transmission and cortical information processing in schizophrenia. Schizophr. Bull..

[47] Agoglia A.E., Sharko A.C., Psilos K.E., Holstein S.E., Reid G.T., Hodge C.W. (2015). Alcohol alters the activation of erk1/2, a functional regulator of binge alcohol drinking in adult c57bl/6j mice. Alcohol. Clin. Exp. Res..

[48] Liu W., Crews F.T. (2015). Adolescent intermittent ethanol exposure enhances ethanol activation of the nucleus accumbens while blunting the prefrontal cortex responses in adult rat. Neuroscience.

[49] Karkhanis A.N., Rose J.H., Huggins K.N., Konstantopoulos J.K., Jones S.R. (2015). Chronic intermittent ethanol exposure reduces presynaptic dopamine neurotransmission in the mouse nucleus accumbens. Drug Alcohol Depend..

[50] Griffin W.C., Ramachandra V.S., Knackstedt L.A., Becker H.C. (2015). Repeated cycles of chronic intermittent ethanol exposure increases basal glutamate in the nucleus accumbens of mice without affecting glutamate transport. Front. Pharmacol..

[51] McGuier N.S., Padula A.E., Mulholland P.J., Chandler L.J. (2015). Homer2 deletion alters dendritic spine morphology but not alcohol-associated adaptations in glun2b-containing *N*-methyl-d-aspartate receptors in the nucleus accumbens. Front. Pharmacol..

[52] Pickering C., Alsio J., Morud J., Ericson M., Robbins T.W., Soderpalm B. (2015). Ethanol impairment of spontaneous alternation behaviour and associated changes in medial prefrontal glutamatergic gene expression precede putative markers of dependence. Pharmacol. Biochem. Behav..

[53] Uys J.D., McGuier N.S., Gass J.T., Griffin W.C., Ball L.E., Mulholland P.J. (2015). Chronic intermittent ethanol exposure and withdrawal leads to adaptations in nucleus accumbens core postsynaptic density proteome and dendritic spines. Addict. Biol..

[54] Peterson V.L., McCool B.A., Hamilton D.A. (2015). Effects of ethanol exposure and withdrawal on dendritic morphology and spine density in the nucleus accumbens core and shell. Brain Res..

[55] Sharma R., Dumontier S., DeRoode D., Sahota P., Thakkar M.M. (2014). Nicotine infusion in the wake-promoting basal forebrain enhances alcohol-induced activation of nucleus accumbens. Alcohol. Clin. Exp. Res..

[56] Ramaker M.J., Strong-Kaufman M.N., Ford M.M., Phillips T.J., Finn D.A. (2014). Effect of nucleus accumbens shell infusions of ganaxolone or gaboxadol on ethanol consumption in mice. Psychopharmacology.

[57] Tizabi Y., Copeland R.L., Louis V.A., Taylor R.E. (2002). Effects of combined systemic alcohol and central nicotine administration into ventral tegmental area on dopamine release in the nucleus accumbens. Alcohol. Clin. Exp. Res..

[58] Kawakami S.E., Quadros I.M., Takahashi S., Suchecki D. (2007). Long maternal separation accelerates behavioural sensitization to ethanol in female, but not in male mice. Behav. Brain Res..

[59] Slotten H.A., Kalinichev M., Hagan J.J., Marsden C.A., Fone K.C. (2006). Long-lasting changes in behavioural and neuroendocrine indices in the rat following neonatal maternal separation: Gender-dependent effects. Brain Res..

[60] Renard G.M., Rivarola M.A., Suarez M.M. (2010). Gender-dependent effects of early maternal separation and variable chronic stress on vasopressinergic activity and glucocorticoid receptor expression in adult rats. Dev. Neurosci..

[61] Cservenka A., Gillespie A.J., Michael P.G., Nagel B.J. (2015). Family history density of alcoholism relates to left nucleus accumbens volume in adolescent girls. J. Stud. Alcohol Drugs.

[62] Del Arco A., Mora F. (2009). Neurotransmitters and prefrontal cortex-limbic system interactions: Implications for plasticity and psychiatric disorders. J. Neural Trans. (Vienna, Aus.: 1996).

[63] O’Donnell P., Grace A.A. (1998). Dysfunctions in multiple interrelated systems as the neurobiological bases of schizophrenic symptom clusters. Schizophr. Bull..

[64] Saddoris M.P., Sugam J.A., Cacciapaglia F., Carelli R.M. (2013). Rapid dopamine dynamics in the accumbens core and shell: Learning and action. Front. Biosci. (Elite ed.).

[65] Bock J., Riedel A., Braun K. (2012). Differential changes of metabolic brain activity and interregional functional coupling in prefronto-limbic pathways during different stress conditions: Functional imaging in freely behaving rodent pups. Front. Cell. Neurosci..

[66] Brake W.G., Zhang T.Y., Diorio J., Meaney M.J., Gratton A. (2004). Influence of early postnatal rearing conditions on mesocorticolimbic dopamine and behavioural responses to psychostimulants and stressors in adult rats. Eur. J. Neurosci..

[67] Oreland S., Raudkivi K., Oreland L., Harro J., Arborelius L., Nylander I. (2011). Ethanol-induced effects on the dopamine and serotonin systems in adult wistar rats are dependent on early-life experiences. Brain Res..

[68] Stamatakis A., Kalpachidou T., Raftogianni A., Zografou E., Tzanou A., Pondiki S., Stylianopoulou F. (2015). Rat dams exposed repeatedly to a daily brief separation from the pups exhibit increased maternal behavior, decreased anxiety and altered levels of receptors for estrogens (eralpha, erbeta), oxytocin and serotonin (5-ht1a) in their brain. Psychoneuroendocrinology.

[69] Kawakami S.E., Quadros I.M., Machado R.B., Suchecki D. (2013). Sex-dependent effects of maternal separation on plasma corticosterone and brain monoamines in response to chronic ethanol administration. Neuroscience.

[70] Greisen M.H., Bolwig T.G., Wortwein G. (2005). Cholecystokinin tetrapeptide effects on hpa axis function and elevated plus maze behaviour in maternally separated and handled rats. Behav. Brain Res..

[71] Plotsky P.M., Meaney M.J. (1993). Early, postnatal experience alters hypothalamic corticotropin-releasing factor (crf) mrna, median eminence crf content and stress-induced release in adult rats. Mol. Brain Res..

[72] Francis D.D., Diorio J., Plotsky P.M., Meaney M.J. (2002). Environmental enrichment reverses the effects of maternal separation on stress reactivity. J. Neurosci..

[73] Nylander I., Roman E. (2013). Is the rodent maternal separation model a valid and effective model for studies on the early-life impact on ethanol consumption?. Psychopharmacology.

[74] Skalkidou A., Hellgren C., Comasco E., Sylven S., Sundstrom Poromaa I. (2012). Biological aspects of postpartum depression. Women’s health.

[75] Bodo C., Rissman E.F. (2006). New roles for estrogen receptor beta in behavior and neuroendocrinology. Front. Neuroendocrinol..

[76] Romens S.E., McDonald J., Svaren J., Pollak S.D. (2015). Associations between early life stress and gene methylation in children. Child Dev..

[77] Oitzl M.S., Champagne D.L., van der Veen R., de Kloet E.R. (2010). Brain development under stress: Hypotheses of glucocorticoid actions revisited. Neurosci. Biobehav. Rev..

[78] Alt S.R., Turner J.D., Klok M.D., Meijer O.C., Lakke E.A., Derijk R.H., Muller C.P. (2010). Differential expression of glucocorticoid receptor transcripts in major depressive disorder is not epigenetically programmed. Psychoneuroendocrinology.

[79] Turecki G., Meaney M.J. (2014). Effects of the social environment and stress on glucocorticoid receptor gene methylation: A systematic review. Biological psychiatry.

[80] Meaney M.J., Szyf M. (2005). Environmental programming of stress responses through DNA methylation: Life at the interface between a dynamic environment and a fixed genome. Dialogues Clin. Neurosci..

[81] Weaver I.C. (2009). Epigenetic effects of glucocorticoids. Semin. Fetal Neonatal Med..

[82] Fish E.W., Shahrokh D., Bagot R., Caldji C., Bredy T., Szyf M., Meaney M.J. (2004). Epigenetic programming of stress responses through variations in maternal care. Ann. NY Acad. Sci..

[83] Witzmann S.R., Turner J.D., Meriaux S.B., Meijer O.C., Muller C.P. (2012). Epigenetic regulation of the glucocorticoid receptor promoter 1(7) in adult rats. Epigenetics.

[84] Schroeder M., Krebs M.O., Bleich S., Frieling H. (2010). Epigenetics and depression: Current challenges and new therapeutic options. Curr. Opin. Psychiatry.

[85] McGowan P.O., Kato T. (2008). Epigenetics in mood disorders. Environ. Health Prev. Med..

[86] McGowan P.O. (2013). Epigenomic mechanisms of early adversity and hpa dysfunction: Considerations for ptsd research. Front. Psychiatry.

[87] Van der Knaap L.J., Riese H., Hudziak J.J., Verbiest M.M., Verhulst F.C., Oldehinkel A.J., van Oort F.V. (2014). Glucocorticoid receptor gene (nr3c1) methylation following stressful events between birth and adolescence. The trails study. Transl. Psychiatry.

[88] De Kloet E.R., Fitzsimons C.P., Datson N.A., Meijer O.C., Vreugdenhil E. (2009). Glucocorticoid signaling and stress-related limbic susceptibility pathway: About receptors, transcription machinery and microrna. Brain Res..

[89] Sofuoglu M., Mooney M. (2009). Cholinergic functioning in stimulant addiction: Implications for medications development. CNS Drugs.

[90] Avena N.M., Rada P.V. (2012). Cholinergic modulation of food and drug satiety and withdrawal. Physiol. Behav..

[91] Mark G.P., Shabani S., Dobbs L.K., Hansen S.T. (2011). Cholinergic modulation of mesolimbic dopamine function and reward. Physiol. Behav..

[92] Pereira P.A., Neves J., Vilela M., Sousa S., Cruz C., Madeira M.D. (2014). Chronic alcohol consumption leads to neurochemical changes in the nucleus accumbens that are not fully reversed by withdrawal. Neurotoxicol. Teratol..

[93] Salamone J.D., Correa M. (2012). The mysterious motivational functions of mesolimbic dopamine. Neuron.

[94] Levita L., Dalley J.W., Robbins T.W. (2002). Nucleus accumbens dopamine and learned fear revisited: A review and some new findings. Behav. Brain Res..

[95] Floresco S.B. (2015). The nucleus accumbens: An interface between cognition, emotion, and action. Ann. Rev. Psychol..

[96] Carlezon W.A., Thomas M.J. (2009). Biological substrates of reward and aversion: A nucleus accumbens activity hypothesis. Neuropharmacology.

[97] Picciotto M.R., Higley M.J., Mineur Y.S. (2012). Acetylcholine as a neuromodulator: Cholinergic signaling shapes nervous system function and behavior. Neuron.

[98] Mesulam M.M., Mufson E.J., Levey A.I., Wainer B.H. (1983). Cholinergic innervation of cortex by the basal forebrain: Cytochemistry and cortical connections of the septal area, diagonal band nuclei, nucleus basalis (substantia innominata), and hypothalamus in the rhesus monkey. J. Comp. Neurol..

[99] Rye D.B., Wainer B.H., Mesulam M.M., Mufson E.J., Saper C.B. (1984). Cortical projections arising from the basal forebrain: A study of cholinergic and noncholinergic components employing combined retrograde tracing and immunohistochemical localization of choline acetyltransferase. Neuroscience.

[100] Blomqvist O., Ericson M., Engel J.A., Soderpalm B. (1997). Accumbal dopamine overflow after ethanol: Localization of the antagonizing effect of mecamylamine. Eur. J. Pharmacol..

[101] Soderpalm B., Lof E., Ericson M. (2009). Mechanistic studies of ethanol’s interaction with the mesolimbic dopamine reward system. Pharmacopsychiatry.

[102] Doyon W.M., Dong Y., Ostroumov A., Thomas A.M., Zhang T.A., Dani J.A. (2013). Nicotine decreases ethanol-induced dopamine signaling and increases self-administration via stress hormones. Neuron.

[103] Ericson M., Blomqvist O., Engel J.A., Soderpalm B. (1998). Voluntary ethanol intake in the rat and the associated accumbal dopamine overflow are blocked by ventral tegmental mecamylamine. Eur. J. Pharmacol..

[104] Larsson A., Edstrom L., Svensson L., Soderpalm B., Engel J.A. (2005). Voluntary ethanol intake increases extracellular acetylcholine levels in the ventral tegmental area in the rat. Alcohol Alcohol..

[105] Gotti C., Zoli M., Clementi F. (2006). Brain nicotinic acetylcholine receptors: Native subtypes and their relevance. Trends Pharmacol. Sci..

[106] Mansvelder H.D., Keath J.R., McGehee D.S. (2002). Synaptic mechanisms underlie nicotine-induced excitability of brain reward areas. Neuron.

[107] Soderpalm B., Ericson M. (2013). Neurocircuitry involved in the development of alcohol addiction: The dopamine system and its access points. Curr. Top. Behav. Neurosci..

[108] Tabakoff B., Hoffman P.L. (2013). The neurobiology of alcohol consumption and alcoholism: An integrative history. Pharmacol. Biochem. Behav..

[109] Hopur52009. Nicotinic_receptors.Png. http://upload.wikimedia.org/wikipedia/commons/f/f1/Nicotinic_receptors.png.

[110] Josephine T.M.S., Holgate J., Bartlett S.E. (2014). Effects of alcohol on nicotinic acetylcholine receptors and impact on addiction. Neuropathol. Addict..

[111] Zaborszky L., Hoemke L., Mohlberg H., Schleicher A., Amunts K., Zilles K. (2008). Stereotaxic probabilistic maps of the magnocellular cell groups in human basal forebrain. NeuroImage.

[112] Zaborszky L., Pang K., Somogyi J., Nadasdy Z., Kallo I. (1999). The basal forebrain corticopetal system revisited. Ann. NY Acad. Sci..

[113] Rahman S., Prendergast M.A. (2012). Cholinergic receptor system as a target for treating alcohol abuse and dependence. Recent Pat. CNS Drug Discov..

[114] Feduccia A.A., Chatterjee S., Bartlett S.E. (2012). Neuronal nicotinic acetylcholine receptors: Neuroplastic changes underlying alcohol and nicotine addictions. Front. Mol. Neurosci..

[115] Çelik T., Kayir H., Ceyhan M., Demirtaş S., Coşar A., Uzbay I.T. (2004). Cpp and amlodipine alter the decrease in basal acetylcholine and choline release by audiogenic stimulus in hippocampus of ethanol-withdrawn rats *in vivo*. Brain Res. Bull..

[116] Casamenti F., Scali C., Vannucchi M.G., Bartolini L., Pepeu G. (1993). Long-term ethanol consumption by rats: Effect on acetylcholine release in vivo, choline acetyltransferase activity, and behavior. Neuroscience.

[117] Mitchell J.M., Teague C.H., Kayser A.S., Bartlett S.E., Fields H.L. (2012). Varenicline decreases alcohol consumption in heavy-drinking smokers. Psychopharmacology.

[118] Chatterjee S., Steensland P., Simms J.A., Holgate J., Coe J.W., Hurst R.S., Shaffer C.L., Lowe J., Rollema H., Bartlett S.E. (2011). Partial agonists of the alpha3beta4* neuronal nicotinic acetylcholine receptor reduce ethanol consumption and seeking in rats. Neuropsychopharmacology.

[119] Steensland P., Simms J.A., Holgate J., Richards J.K., Bartlett S.E. (2007). Varenicline, an alpha4beta2 nicotinic acetylcholine receptor partial agonist, selectively decreases ethanol consumption and seeking. Proc. Natl. Acad. Sci. USA.

[120] Kuzmin A., Jerlhag E., Liljequist S., Engel J. (2009). Effects of subunit selective nach receptors on operant ethanol self-administration and relapse-like ethanol-drinking behavior. Psychopharmacology.

[121] Reuter M., Markett S., Melchers M., Montag C. (2012). Interaction of the cholinergic system and the hypothalamic-pituitary-adrenal axis as a risk factor for depression: Evidence from a genetic association study. Neuroreport.

[122] Aisa B., Gil-Bea F.J., Marcos B., Tordera R., Lasheras B., del Rio J., Ramirez M.J. (2009). Neonatal stress affects vulnerability of cholinergic neurons and cognition in the rat: Involvement of the hpa axis. Psychoneuroendocrinology.

[123] Okada S., Yamaguchi-Shima N., Shimizu T., Arai J., Lianyi L., Wakiguchi H., Yokotani K. (2008). Role of brain nicotinic acetylcholine receptor in centrally administered corticotropin-releasing factor-induced elevation of plasma corticosterone in rats. European Journal of Pharmacol..

[124] Loomis S., Gilmour G. (2010). Corticosterone urinalysis and nicotinic receptor modulation in rats. J. Neurosci. Methods.

[125] Kim S.A., Kim J.W., Song J.Y., Park S., Lee H.J., Chung J.H. (2004). Association of polymorphisms in nicotinic acetylcholine receptor alpha 4 subunit gene (chrna4), mu-opioid receptor gene (oprm1), and ethanol-metabolizing enzyme genes with alcoholism in korean patients. Alcohol.

[126] Schulz K.M., Andrud K.M., Burke M.B., Pearson J.N., Kreisler A.D., Stevens K.E., Leonard S., Adams C.E. (2013). The effects of prenatal stress on alpha4 beta2 and alpha7 hippocampal nicotinic acetylcholine receptor levels in adult offspring. Dev. Neurobiol..

[127] Cservenka A., Casimo K., Fair D.A., Nagel B.J. (2014). Resting state functional connectivity of the nucleus accumbens in youth with a family history of alcoholism. Psychiatry Res..

[128] Weiland B.J., Welsh R.C., Yau W.Y., Zucker R.A., Zubieta J.K., Heitzeg M.M. (2013). Accumbens functional connectivity during reward mediates sensation-seeking and alcohol use in high-risk youth. Drug Alcohol Depend..

[129] Andrews M.M., Meda S.A., Thomas A.D., Potenza M.N., Krystal J.H., Worhunsky P., Stevens M.C., O’Malley S., Book G.A., Reynolds B. (2011). Individuals family history positive for alcoholism show functional magnetic resonance imaging differences in reward sensitivity that are related to impulsivity factors. Biol. Psychiatry.

[130] Markett S., Montag C., Reuter M. (2011). The nicotinic acetylcholine receptor gene chrna4 is associated with negative emotionality. Emotion (Washington, DC).

[131] Cippitelli A., Brunori G., Gaiolini K.A., Zaveri N.T., Toll L. (2015). Pharmacological stress is required for the anti-alcohol effect of the alpha3beta4* nachr partial agonist at-1001. Neuropharmacology.

[132] Baier C.J., Pallares M.E., Adrover E., Monteleone M.C., Brocco M.A., Barrantes F.J., Antonelli M.C. (2015). Prenatal restraint stress decreases the expression of alpha-7 nicotinic receptor in the brain of adult rat offspring. Stress (Amst., Neth.).

[133] Taslim N., Soderstrom K., Dar M.S. (2011). Role of mouse cerebellar nicotinic acetylcholine receptor (nachr) alpha(4)beta(2)- and alpha(7) subtypes in the behavioral cross-tolerance between nicotine and ethanol-induced ataxia. Behav. Brain Res..

[134] Pichat P., Bergis O.E., Terranova J.P., Urani A., Duarte C., Santucci V., Gueudet C., Voltz C., Steinberg R., Stemmelin J. (2007). Ssr180711, a novel selective alpha7 nicotinic receptor partial agonist: (ii) Efficacy in experimental models predictive of activity against cognitive symptoms of schizophrenia. Neuropsychopharmacology.

[135] Robles N., Sabria J. (2006). Ethanol consumption produces changes in behavior and on hippocampal alpha7 and alpha4beta2 nicotinic receptors. J. Mol. Neurosci. MN.

